# Supplementary material for: Simian Foamy Virus Prevalence and Evolutionary Relationships in Two Free-Living Lion Tamarin Populations from Rio de Janeiro, Brazil
Source: Viruses. 2025 Jul 31;17(8):1072. doi: 10.3390/v17081072 (PMC12390735; doi:10.3390/v17081072)

Supplementary Table S1. SFV prevalence and viral load according to the location of data collection to *Leontopithecus rosalia*.

Collection point	Group	Animals	Prevalence (%)	Mean proviral load*
Afetiva	Afetiva 1	2	50%%	2.05
Afetiva	Afetiva 2/ AF2	12	25%	2.19
Afetiva	Afetiva 3/ AF3	2	0%	N/A**
Afetiva	UR	4	0%	N/A
Afetiva	FP	5	20%	2.33
Afetiva	FP3	1	0%	N/A
Andorinha	CH2	3	100%	4.22
Tamarins	Sidney 3	1	100%	3.49
Tamarins	TM2	4	25,00%	4.12
Igarapé	IG	8	62%	2.81
Igarapé	ph2	4	25%	1.88
Moriá Mount	Ronaldo Machado (RM)	2	40%	6.63
Nova Esperança	GM2	3	0%	N/A
Nova Esperança	GM3	7	29%	3.02
Nova Esperança	GM4	2	100%	3.05
Nova Esperança	GM5	4	0%	N/A
Nova Esperança	GM7	3	0%	N/A
Rio vermelho	M6	1	100%	2.51
Rio vermelho	Mistura fina	3	0%	N/A
Rio vermelho	RV	4	25%	2.96
Rio vermelho	RT	1	0%	N/A
Ribeirão	ZN	2	0%	N/A
Santa Helena	FN	2	100%	2.65
Santa Helena	JA	5	20%	4.34
Santa Helena	JN	2	100%	4.30
Santa Helena	JR	4	0%	N/A

Santa Helena 1	SH	1	0%	N/A
Santa Helena 1	SS2	3	67%	4.56
Sítio Quelinho	q1	2	100%	3.91
Tertulio	JD	2	50%	3.93

*Mean viral load (log_{10}) per 10^6 cells

** N/A = not available

Supplementary Table S2. SFV sequences used to characterize the novel SFVlro.

Accession	Host	Host genus	Annotation	Animal status
NC_039027.1	*Ateles sp.*	*Ateles*	Atelidae	Captive
NC_039030.1	*Callithrix*	*Callithrix*	Callitrichidae	Captive
NC_039031.1	*Sapajus xanthosternos*	*Sapajus*	Cebidae	Captive
LC487610.1	*Macaca fuscata*	*Macaca*	Cercopithecidae	Captive
LC487611.1	*Macaca fuscata yakui*	*Macaca*	Cercopithecidae	Captive
LC487615.1	*Macaca fuscata*	*Macaca*	Cercopithecidae	Captive
LC487619.1	*Macaca mulatta*	*Macaca*	Cercopithecidae	Captive
LC487620.1	*Macaca cyclopis*	*Macaca*	Cercopithecidae	Captive
LC487623.1	*Macaca fuscata yakui*	*Macaca*	Cercopithecidae	Captive
LC487624.1	*Macaca fuscata yakui*	*Macaca*	Cercopithecidae	Captive
LC487626.1	*Macaca fuscata yakui*	*Macaca*	Cercopithecidae	Captive
MN178627.1	*Leontopithecus chrysomelas*	*Leontopithecus*	Callitrichidae	Captive
MN178628.1	*Leontopithecus chrysomelas*	*Leontopithecus*	Callitrichidae	Captive
MN178629.1	*Leontopithecus chrysomelas*	*Leontopithecus*	Callitrichidae	Captive
MN178630.1	*Leontopithecus chrysomelas*	*Leontopithecus*	Callitrichidae	Captive
MN178631.1	*Leontopithecus*	*Leontopithecus*	Callitrichidae	Captive

	chrysomelas			
MN178632.1	*Leontopithecus chrysomelas*	*Leontopithecus*	Callitrichidae	Captive
MN178633.1	*Leontopithecus chrysomelas*	*Leontopithecus*	Callitrichidae	Captive
MN178635.1	*Leontopithecus chrysomelas*	*Leontopithecus*	Callitrichidae	Captive
MN178636.1	*Leontopithecus chrysomelas*	*Leontopithecus*	Callitrichidae	Captive
MH368762.1	*Brachyteles arachnoides*	*Brachyteles*	Atelidae	Captive
KR528435.1	*Sapajus xanthosternos*	*Sapajus*	Cebidae	Captive
KR528436.1	*Sapajus nigritus robustus*	*Sapajus*	Cebidae	Captive
KR528438.1	*Cacajao melanocephalus*	*Cacajao*	Pitheciidae	Captive
KR528439.1	*Sapajus nigritus robustus*	*Sapajus*	Cebidae	Captive
KR528442.1	*Alouatta guariba*	*Alouatta*	Atelidae	Captive
KR528443.1	*Leontopithecus chrysomelas*	*Leontopithecus*	Callitrichidae	Captive
KR528444.1	*Callithrix geoffroyi*	*Callithrix*	Callitrichidae	Captive
KR528445.1	*Chiropotes sp.*	*Chiropotes*	Pitheciidae	Captive
KR528447.1	*Alouatta belzebul*	*Alouatta*	Atelidae	Captive
KR902438.1	*Ateles chamek*	*Ateles*	Atelidae	Free living
KR902443.1	*Ateles geoffroyi*	*Ateles*	Atelidae	Captive
KR902444.1	*Ateles geoffroyi*	*Ateles*	Atelidae	Captive
KR902448.1	*Ateles geoffroyi*	*Ateles*	Atelidae	Captive
KR902451.1	*Ateles hybridus*	*Ateles*	Atelidae	Captive
KR902454.1	*Alouatta sara*	*Alouatta*	Atelidae	Captive
KR902456.1	*Alouatta seniculus*	*Alouatta*	Atelidae	Captive
KR902458.1	*Sapajus apella*	*Sapajus*	Cebidae	Captive
KR902459.1	*Sapajus apella*	*Sapajus*	Cebidae	Captive

KR902460.1	*Sapajus apella*	*Sapajus*	Cebidae	Captive
KR902461.1	*Sapajus apella*	*Sapajus*	Cebidae	Captive
KR902464.1	*Sapajus apella*	*Sapajus*	Cebidae	Captive
KR902465.1	*Sapajus apella*	*Sapajus*	Cebidae	Captive
KR902466.1	*Sapajus apella*	*Sapajus*	Cebidae	Captive
KR902470.1	*Sapajus apella*	*Sapajus*	Cebidae	Captive
KR902473.1	*Callithrix jacchus*	*Callithrix*	Callitrichidae	Captive
KR902481.1	*Pithecia pithecia*	*Pithecia*	Pitheciidae	Captive
KR902483.1	*Pithecia pithecia*	*Pithecia*	Pitheciidae	Captive
KR902490.1	*Callithrix jacchus*	*Callithrix*	Callitrichidae	Captive
KC283230.1	*Macaca fascicularis*	*Macaca*	Cercopithecidae	Free living
KC283231.1	*Macaca fascicularis*	*Macaca*	Cercopithecidae	Free living
KC283234.1	*Macaca fascicularis*	*Macaca*	Cercopithecidae	Free living
KC283236.1	*Macaca fascicularis*	*Macaca*	Cercopithecidae	Free living
KC196056.1	*Macaca mulatta*	*Macaca*	Cercopithecidae	Captive
KC196057.1	*Macaca mulatta*	*Macaca*	Cercopithecidae	Captive
KC196058.1	*Macaca mulatta*	*Macaca*	Cercopithecidae	Captive
KC196059.1	*Macaca mulatta*	*Macaca*	Cercopithecidae	Captive
KC331074.1	*Alouatta seniculus*	*Alouatta*	Atelidae	Captive
KC331075.1	*Sapajus albifrons*	*Sapajus*	Cebidae	Captive
KC331077.1	*Sapajus apella*	*Sapajus*	Cebidae	Captive
KC331078.1	*Sapajus apella*	*Sapajus*	Cebidae	Captive
KC331079.1	*Sapajus apella*	*Sapajus*	Cebidae	Captive
KC331080.1	*Sapajus apella*	*Sapajus*	Cebidae	Captive
KC331081.1	*Sapajus xanthosternos*	*Sapajus*	Cebidae	Captive
KC331082.1	*Alouatta guariba*	*Alouatta*	Atelidae	Captive
JF746869.1	*Macaca mulatta*	*Macaca*	Cercopithecidae	Captive
EU527595.1	*Pan paniscus*	*Pan*	Hominidae	Free living
DQ354074.1	*Macaca tonkeana*	*Macaca*	Cercopithecidae	Captive

DQ354080.1	*Macaca tonkeana*	*Macaca*	Cercopithecidae	Captive
AY686195.1	*Pan paniscus*	*Pan*	Hominidae	Captive
AY686198.1	*Macaca arctoides*	*Macaca*	Cercopithecidae	Captive
AJ627527.1	*Pongo pygmaeus pygmaeus*	*Pongo*	Hominidae	N/A*
AJ627528.1	*Pongo pygmaeus pygmaeus*	*Pongo*	Hominidae	N/A
AJ627531.1	*Pongo pygmaeus pygmaeus*	*Pongo*	Hominidae	N/A
AJ627533.1	*Pongo pygmaeus pygmaeus*	*Pongo*	Hominidae	N/A
AJ627534.1	*Pongo pygmaeus pygmaeus*	*Pongo*	Hominidae	N/A
AJ627536.1	*Pongo pygmaeus pygmaeus*	*Pongo*	Hominidae	N/A
AJ627543.1	*Pongo abelii*	*Pongo*	Hominidae	Captive
AJ627544.1	*Pongo abelii*	*Pongo*	Hominidae	Free living
AJ627547.1	*Pongo abelii*	*Pongo*	Hominidae	Captive
AJ627550.1	*Pan paniscus*	*Pan*	Hominidae	N/A
AJ627551.1	*Pan paniscus*	*Pan*	Hominidae	N/A
AY278785.1	*Cercocebus torquatus*	*Cercocebus*	Cercopithecidae	Human sample
AY195689.1	*Pongo pygmaeus*	*Pongo*	Hominidae	Human sample
AJ556783.1	*Pongo pygmaeus pygmaeus*	*Pongo*	Hominidae	Captive
AF516486.1	*Hylobates pileatus*	*Hylobates*	Hylobatidae	Captive
AF516487.1	*Nomascus leucogenys*	*Nomascus*	Hylobatidae	Captive
AF049086.1	*Pongo pygmaeus*	*Pongo*	Hominidae	Free living
X83298.1	*Ateles sp.*	*Ateles*	Atelidae	N/A
KR528446.1	*Leontopithecus rosalia*	*Leontopithecus*	Callitrichidae	Captive
PP960560.1	*Leontopithecus rosalia*	*Leontopithecus*	This Study	Free living
NC_039023.1	*Otolemur*	*Otolemur*	Outgroup	Captive

	crassicaudatus
	panganiensis

N/A* = Not available

Supplementary Table S3. Likelihood mapping plots values of used aligned.

Region	Percentage	Interpretation
Corner 1	22.4%	Strong signal for topology A
Corner 2	20%	Strong signal for topology B
Corner 3	20.7%	Strong signal for topology C
Edge 1	1.8%	Partial support between A/B
Edge 2	2.2%	Partial support between B/C
Edge 3	2.1%	Partial support between C/A
Center	30.8%	Unresolved quartets (ambiguity)

Supplementary Table S4. Test of substitution saturation performed in all sites. Two-tailed t-tests are used. Percentage of invariant sites were calculated by building an UPGMA tree under the GTR model, with value of P(invariant) of 0,08403.

NumOTU	Iss	Sym		Asym		Conclusion
		Iss.c	p-value	Iss.c	p-value	
4	0.451	0.756	< 0.0001	0.565	< 0.0001	Little saturation
8	0.452	0.729	< 0.0001	0.634	0.0001	Little saturation
14	0.477	0.648	0,0004	0.456	0.6568	Little saturation*
32	0.489	0.688	0,0001	0.369	0.016	Little saturation

For NumOTU 14 under asymmetrical topology (Iss.c = 0.456), Iss was not significantly lower (p = 0.6568).

Supplementary Table S5. Node dates and confidence intervals of major clades of SFV inferred in this study.

Major SFV Splits	Million Years Ago 95% confidence interval [Lower–Upper]
SFV Leontopithecus rosalia	0.0836 [0.0362 - 0.1931]
SFV Sapajus Strain 1	0.0032 [0 - 0.03588]
SFV Leontopithecus chrysomelas Strain 1	0.0844 [0.021 - 0.3396]
SFV Sapajus Strain 1 & SFV Leontopithecus rosalia	0.7071 [0.3015-1.6858]
SFV Sapajus Strain 1 & SFV Leontopithecus chrysomelas Strain 1	1.1471 [0.5472-2.4046]
SFV Sapajus Strain 2	1.9051 [0.904-3.5752]
SFV Sapajus Strain 1 & 2	3.79 [2.137-6.3811]
SFV Callitrichidae & SFV Cebidae	4.2332 [2.807 - 6.3811]
SFV Sapajus Strain 3	0.2395 [0.1319 - 0.4348]
SFV Sapajus Strain 3 & SFV Leontopithecus chrysomelas Strain 2 & SFV Callithrix	3.652 [2.106 - 6.343]
SFV Callithrix	2.6395 [1.12694 - 5.4884]
SFV Leontopithecus chrysomelas Strain 2	0.0654 [0.0032 - 0.6261]
SFV Leontopithecus chrysomelas Strain 2 & SFV Sapajus nigritus robustus | KR528439.1	2.4733 [1.1895 - 5.1426]
SFV Atelidae	12.1589 [9.0628 - 16.3127]
SFV Platyrrhini & Catarrhini*	39.6367 [38.4684 - 40.65]
SFV Platyrrhini	24.5659 [21.8374 - 27.6354]
SFV Catarrhini*	29.1632 [29.1632 - 30.6852]
SFV Macaca*	6.0542 [5.5918 - 6.1425]
SFV Pongo*	1.8633 [1.6461 - 1.8633]
SFV Pan*	8.024 [7.4887 - 8.705]
SFV Pongo & SFV Pan*	20.8554 [19.6804 - 21.8844]
SFV Pongo & SFV Pan & SFV Pongo	21.3642 [19.6804 - 25.6621]

* Calibration points

Supplementary Figure S1. Likelihood mapping plots of used aligned.

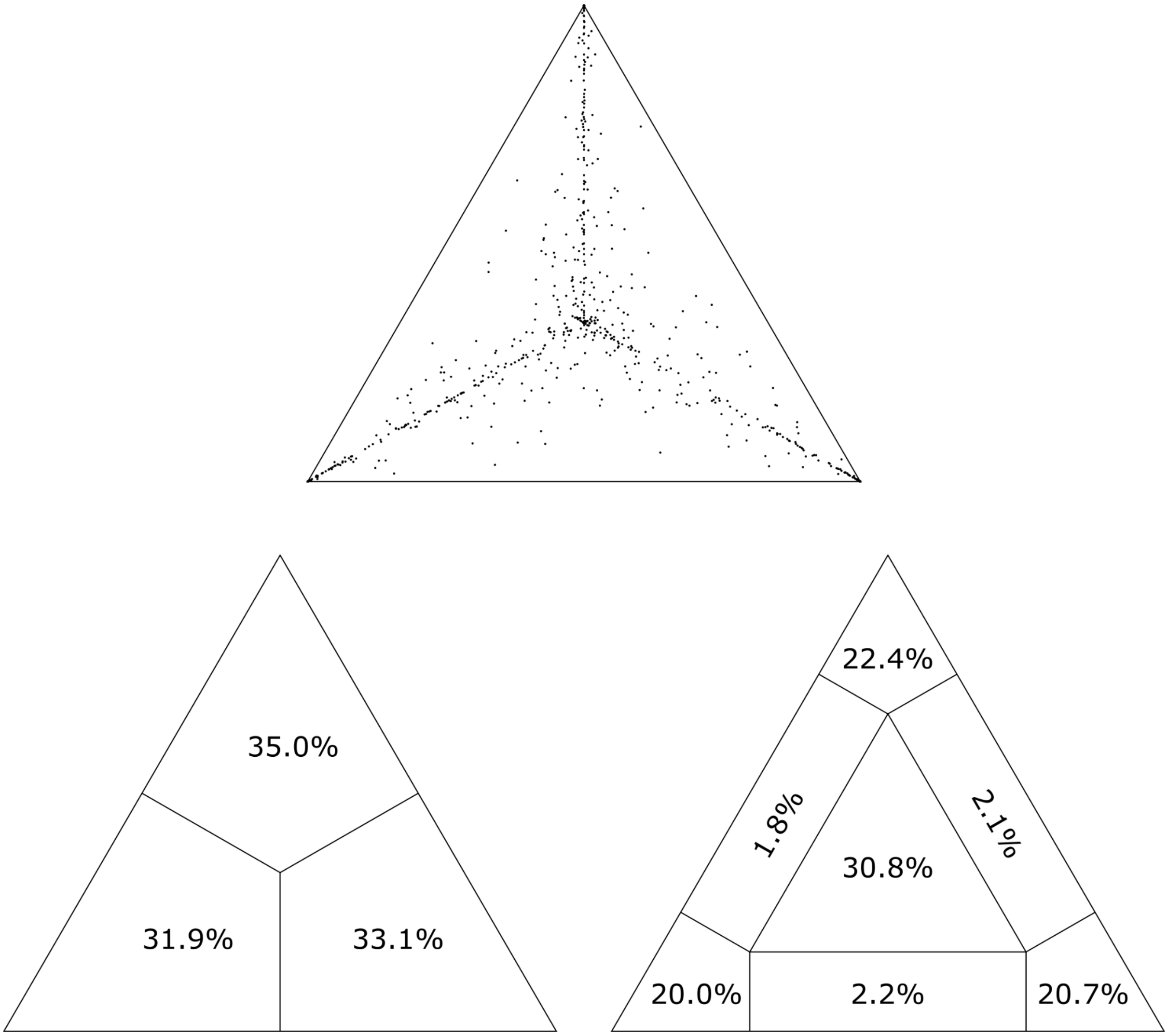

Supplementary Figure S2 - GUIDANCE2 alignment confidence.

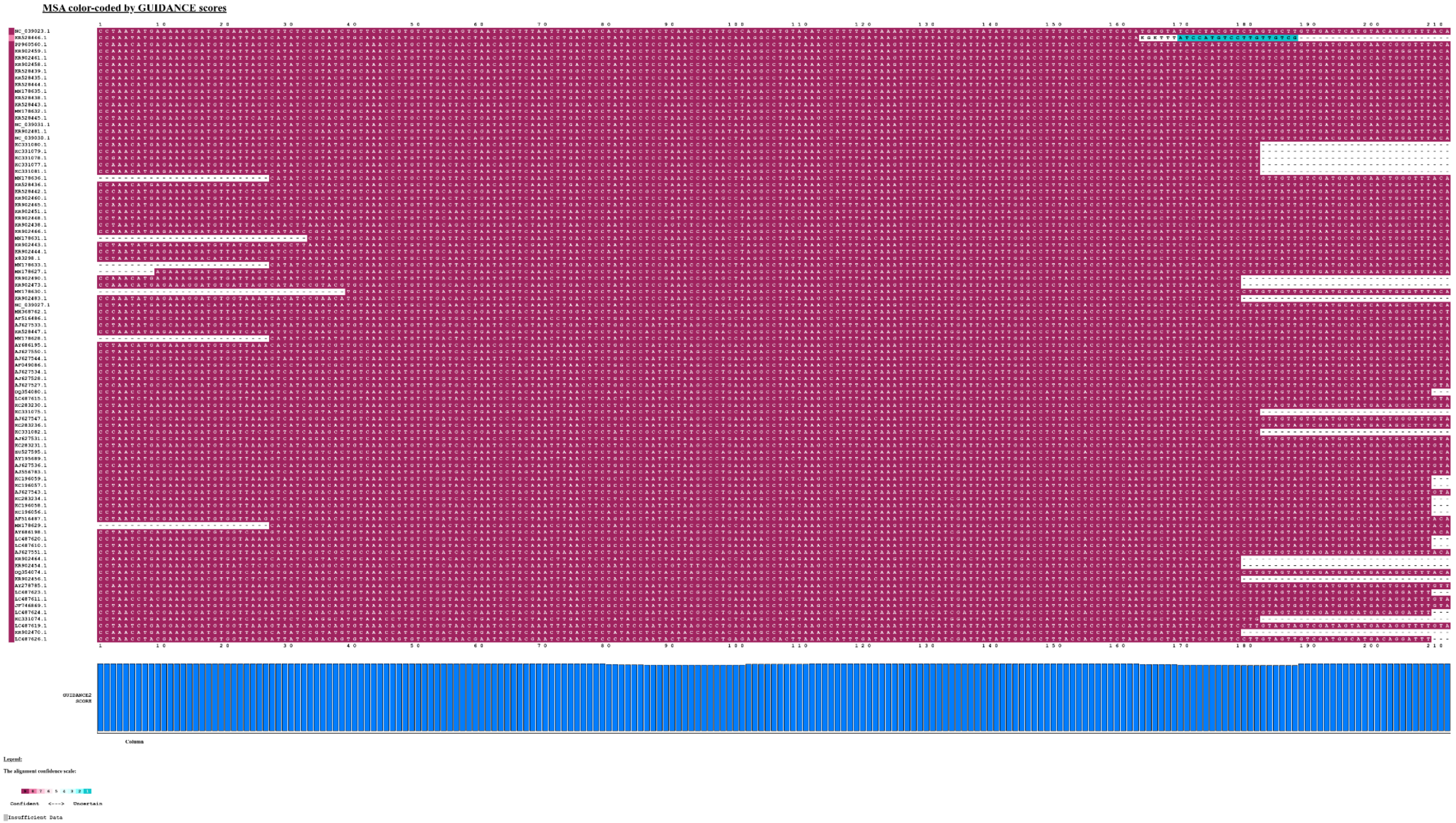

Supplementary Figure S3 - Complete timescale phylogenetic tree generated by RelTime-ML. Estimated host divergence dates were used to calibrate internal nodes of the viral tree. The node labels are colored according to the host family used in the dataset. The sequence generated in the current study is marked with a golden star. The x-axis summarizes the geological time scale of the timetree: Oligocene (Ol), Miocene (Mio), Plioceno (Pli) and Pleistocene (Ple).

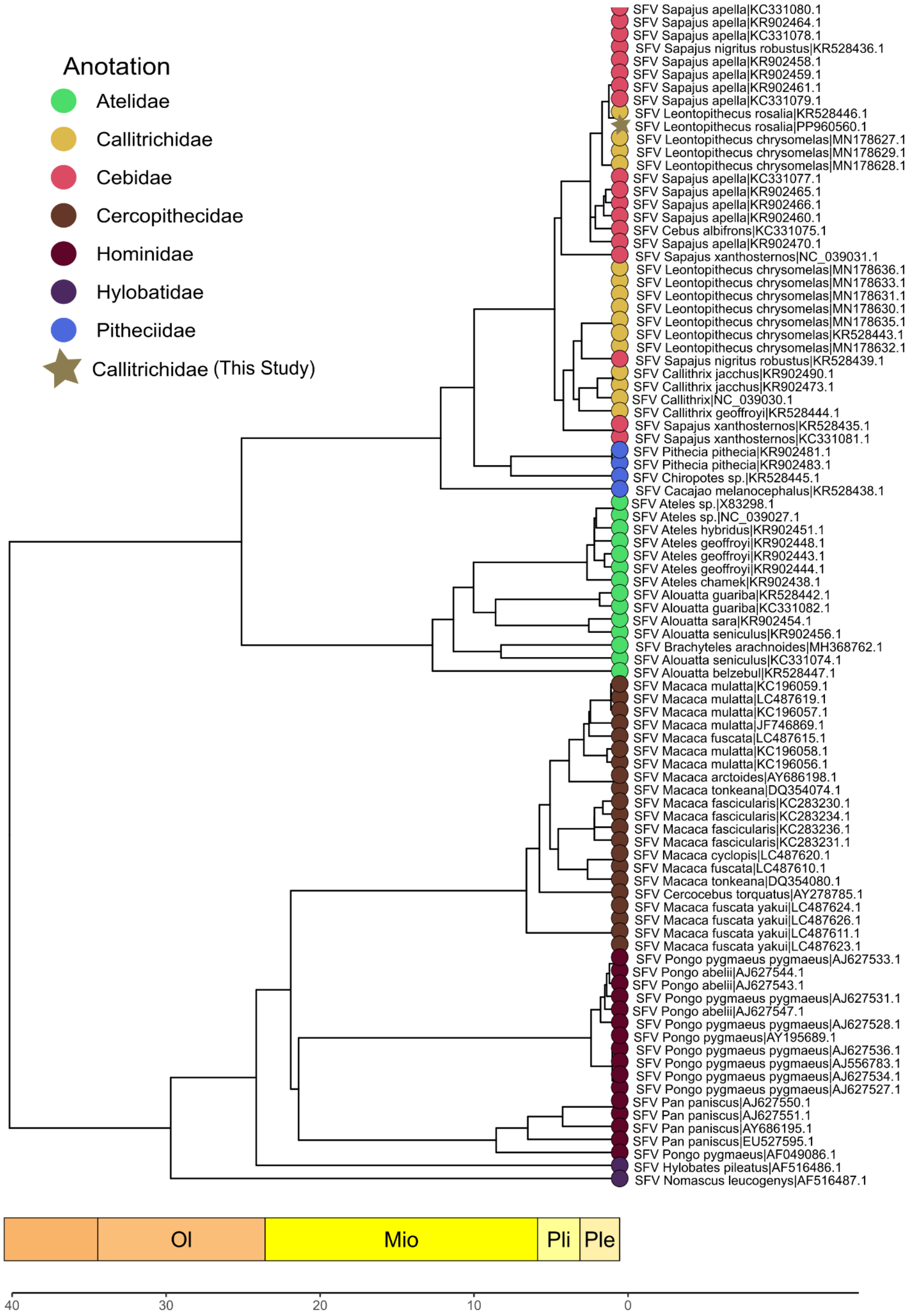

Supplement: Supplementary file 1 [file viruses-17-01072-s001.zip › viruses-3562984-supplementary.pdf]
